# Beyond the Take-Home Pathway: Community-Level Pesticide Exposure Among Children Living in an Intensively Cultivated Agricultural Landscape

**DOI:** 10.3390/ijerph23050664

**Published:** 2026-05-18

**Authors:** Humberto González

**Affiliations:** Centre for Research and Advanced Studies in Social Anthropology (CIESAS), West Unit, Guadalajara Jal. 44630, Mexico; hgc@ciesas.edu.mx

**Keywords:** pesticide exposure, child health, urinary biomonitoring, agricultural communities, community-level exposure regime

## Abstract

**Highlights:**

**Public health relevance—How does this work relate to a public health issue?**

- This study documents pesticide exposure among children in a rural agricultural community in Mexico, addressing a critical and underreported environmental health issue.
- It links environmental contamination with child health risks in contexts of intensive agrochemical use and structural vulnerability.

**Public health significance—Why is this work of significance to public health?**

- The study provides longitudinal biomonitoring evidence of sustained and seasonal pesticide exposure in children, highlighting patterns often overlooked in cross-sectional research.
- It contributes to understanding how agricultural production systems shape cumulative exposure and health risks in vulnerable populations.

**Public health implications—What are the key implications or messages for practitioners, policy makers and/or researchers in public health?**

- The findings underscore the need for stronger regulatory frameworks, surveillance systems, and preventive interventions to reduce children’s exposure to pesticides in rural settings.
- They support the integration of environmental, social, and health strategies—such as agroecological transitions—to address structurally embedded exposure risks.

**Abstract:**

Children living in agricultural regions are exposed to pesticides through multiple environmental and occupational exposure processes; however, the relative contribution of these processes remains insufficiently characterised in many rural contexts of the Global South. This study assessed pesticide exposure among children residing in an agricultural community in western Mexico characterised by close spatial proximity between residential areas and intensively cultivated fields. Urine samples were collected from children at two points in the agricultural cycle (March and December 2018). Pesticide concentrations were determined using liquid chromatography coupled with tandem mass spectrometry (LC–MS/MS). Paired longitudinal analyses were conducted to evaluate intra-individual changes in detection frequencies and urinary concentrations across sampling periods. Multiple pesticides were detected, including compounds with near-universal presence across both sampling periods. Significant increases in urinary concentrations were observed between March and December for several pesticides, consistent with seasonal agricultural dynamics, while no systematic differences were identified between children from agricultural and non-agricultural households. These findings indicate that pesticide exposure in this setting operates as a community-level exposure regime that is both structurally produced and territorially embedded. Exposure patterns reflect the convergence of agricultural practices, environmental dispersion processes, and spatial configurations that extend beyond occupational boundaries. The results highlight the limitations of risk models focused exclusively on individual or occupational exposure and underscore the need for public health strategies that address pesticide exposure as a structurally produced and territorially embedded condition.

## 1. Introduction

Pesticides are widely recognised as hazardous substances because of their harmful effects on human health and ecosystems. Although exposure has traditionally been analysed in relation to agricultural workers, growing evidence shows that children living in both rural and urban settings may also be exposed, even when they are not directly involved in agricultural activities [1,2]. Exposure is not limited to direct contact during application but may occur through multiple pathways, including spray drift, contaminated soil and dust, water, food, and residues transported into domestic environments.

In recent years, a growing body of research has substantially advanced the understanding of pesticide exposure in children, particularly through biomonitoring and longitudinal cohort studies. Evidence from European and international cohorts has demonstrated associations between exposure to non-persistent pesticides and developmental outcomes, including pubertal timing and neurodevelopmental processes [3]. Complementary biomonitoring studies conducted in diverse agricultural settings, including Latin America, have reported consistently elevated levels of pesticide metabolites among children and adolescents, highlighting the persistence and widespread nature of exposure across different agricultural production systems [4,5].

Recent advances in analytical methodologies have facilitated the simultaneous assessment of multiple urinary pesticide metabolites and biomarkers of oxidative stress, offering more comprehensive insights into pesticide exposure and associated biological responses [6]. Together, these studies establish a robust empirical foundation for situating local findings within a broader global context of widespread and unevenly distributed pesticide exposure in childhood.

Among these pathways, the environmental dispersion of pesticides beyond application sites is particularly relevant in agricultural settings, where treated fields, homes, schools, and public spaces often coexist in close spatial proximity [7]. This issue is especially significant in rural populations, where agriculture remains a dominant economic activity and where large numbers of people live in close contact with agricultural landscapes. In Mexico, rural localities account for a substantial share of the population, and a significant proportion of economically active residents are engaged in primary activities such as agriculture [8]. These conditions create environments in which agricultural production and everyday life are closely intertwined.

Recent research has further highlighted the complexity of exposure processes associated with contemporary pesticide use, underscoring that toxicity is shaped not only by active ingredients but also by co-formulants and adjuvants that influence environmental persistence and bioavailability [9]. Furthermore, contemporary pesticide classes such as neonicotinoids have been linked to developmental neurotoxicity. This evidence raises significant concerns regarding their pervasive use and the potential long-term implications for paediatric health [10]. Emerging evidence also underscores the importance of indirect exposure processes, particularly pesticide drift associated with aerial or mechanised spraying, which extends exposure beyond occupational settings and into surrounding communities [11]. Taken together, these findings point to the need to conceptualise exposure as a diffuse and spatially distributed process rather than as a confined occupational risk.

In parallel, recent scholarship in environmental health, environmental justice, and socio-environmental research has increasingly emphasised the importance of community-level exposure as a key analytical framework. Studies conducted in low- and middle-income countries have shown that children’s exposure to pesticides is shaped by structural conditions, including agricultural intensification, regulatory gaps, and socio-economic vulnerability [12]. Conceptual developments in this area stress that exposure is not only individual but also territorially embedded, emerging from the interaction between environmental contamination, labour dynamics, and everyday living conditions [13]. From this perspective, pesticide exposure becomes a collective and structurally mediated phenomenon, closely linked to processes of environmental injustice [14], and requiring integrative approaches that connect health, territory, and socio-economic organisation.

Children constitute a population of particular concern because of their heightened biological and behavioural susceptibility to environmental contaminants. During early life, exposure relative to body weight is higher, detoxification systems are still developing, and behaviours such as crawling and hand-to-mouth activity increase contact with contaminated surfaces and residues. These factors make childhood a particularly sensitive period in relation to environmental toxicants and their potential short- and long-term health effects [15,16,17,18].

Most studies of children’s exposure in agricultural regions have emphasised occupationally mediated routes, particularly the take-home pathway, through which residues are transported from the workplace to the household on workers’ clothing, footwear, skin, or equipment. While this mechanism is well-established, it does not fully explain the exposure patterns observed in many rural communities, where children from non-farming households may also present measurable pesticide exposure [19,20,21,22]. This suggests that pesticide exposure in such settings should be understood not only as an occupational problem, but also as a broader socio-environmental process shaped by environmental dispersion, residential proximity to treated fields, and the territorial organisation of agricultural production.

These limitations point to the need for a broader analytical framework capable of capturing the complexity of exposure in agricultural settings. In this study, pesticide exposure is conceptualised as a community-level exposure regime in which diverse environmental and socio-spatial processes converge and are continuously reproduced within a shared territorial space. From this perspective, exposure is understood as structurally produced through agricultural systems, labour arrangements, and regulatory conditions, and as territorially embedded in landscapes where residential and productive spaces are closely intertwined. This approach allows for a more comprehensive interpretation of children’s exposure, moving beyond individual or household-level explanations toward a recognition of exposure as a socially and spatially distributed process.

This study adopts a transdisciplinary perspective to examine children’s exposure to pesticides in a rural community in western Mexico [23]. It integrates approaches and methods from the health sciences, environmental sciences, and social sciences in order to analyse exposure not only as a biological event, but also as a socio-environmental phenomenon embedded in place. In analytical terms, the study draws on a multi-scalar perspective that links local exposure conditions with wider historical and structural processes shaping rural health and environmental vulnerability [24].

Among these processes, the agro-industrial production model is particularly important. Consolidated in Mexico during the Green Revolution and later expanded through global agri-food restructuring, this model promoted monoculture expansion, technological intensification, and the systematic use of agrochemicals to sustain productivity and competitiveness [25,26,27]. Although this model increased agricultural output, it also deepened ecological degradation, technological dependency, and health risks in rural territories where intensive production became the dominant form of land use [28,29]. From this perspective, children’s exposure to pesticides is not merely the result of isolated practices, but part of a broader socio-environmental configuration in which the costs of production are externalised onto rural populations and landscapes [30,31].

**Figure 1:**
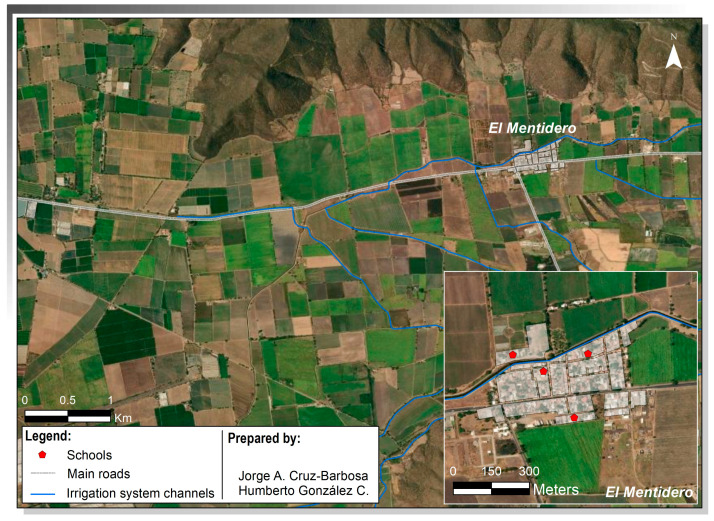
El Mentidero is located within the agricultural valley of Autlán, El Grullo and El Limón in the state of Jalisco [34].

These broader agro-industrial transformations are not abstract processes but are materialised in specific rural territories, where patterns of land use, labour organisation, and settlement shape everyday exposure conditions. These dynamics are concretely expressed in El Mentidero (EM) (Figure 1), a rural locality in western Mexico with a population of 1399 inhabitants [32] situated within an intensively cultivated agricultural valley dominated by sugarcane and horticultural production [33]. Agriculture constitutes the main economic activity in the region and has historically driven both land use and population dynamics.

The demographic configuration of the locality has been shaped by the long-term settlement of agricultural labourer families, together with ongoing seasonal migration linked to crop cycles. Since the expansion of irrigation infrastructure and intensive agriculture in the late twentieth century, EM has experienced sustained in-migration of rural workers, many of whom reside temporarily under precarious housing conditions associated with agricultural labour [35,36]. These patterns of mobility and settlement have produced a socially and spatially heterogeneous environment characterised by conditions of labour, residential, and environmental vulnerability.

In this context, the close proximity between residential areas, temporary settlements, and cultivated fields is a defining characteristic of the study setting. In many cases, households are located at distances ranging from only a few metres to approximately 100 m from actively treated agricultural plots (Figure 1). This spatial configuration, in which living spaces are directly embedded within areas of intensive pesticide application, creates conditions that facilitate continuous environmental exposure. These dynamics are reflected in the everyday interaction between domestic, school, community, and agricultural spaces, making El Mentidero a particularly relevant setting for examining how pesticide exposure is produced and reproduced in daily life.

Against this background, the aim of this study was to assess pesticide exposure among children living in this agricultural community through longitudinal urinary biomonitoring and to examine how such exposure is produced within a community-level exposure regime shaped by environmental dispersion, agricultural production systems, and territorial organisation. Rather than assuming differential exposure based on household occupation, the study explores the extent to which exposure operates as a community-wide condition. In this context, a community-level pesticide exposure regime refers to a structurally produced and territorially embedded system through which exposure to agrochemicals extends beyond individual or occupational processes and becomes a collective phenomenon affecting the entire community. This regime is characterised by the spatial diffusion of pesticides across residential and public spaces, temporal patterns linked to agricultural cycles, and generalised exposure irrespective of individuals’ direct involvement in agricultural activities.

By combining biomonitoring, socio-agronomic information, and ethnographic evidence, the study contributes to a more comprehensive understanding of children’s exposure to pesticides in agricultural regions and informs public health approaches that move beyond narrowly occupational models of risk.

## 2. Materials and Methods

### 2.1. Study Design and Participants

This study combined three complementary methodological strategies: (i) longitudinal biomonitoring of urinary pesticide concentrations in schoolchildren; (ii) administration of structured questionnaires to heads of households to collect socio-agronomic information; and (iii) ethnographic fieldwork to examine the socio-demographic context and everyday exposure conditions of children living in the study community.

In the first phase of the study, contact was established with the directors of the local kindergarten and primary schools, and through them, with parents or guardians to explain the study objectives and request participation. Written informed consent was obtained from all participating parents or guardians, and the confidentiality of both survey information and laboratory results was ensured. The study was approved by the Ethics Committee of the Department of Public Health at the University of Guadalajara (approval number: DCSP/CEI/2016/260618/038).

The study initially recruited 101 schoolchildren through simple random sampling. Urine samples were collected in two field campaigns conducted in March and December 2018. Due to incomplete participation across sampling rounds and the exclusion of unpaired samples, the analytical longitudinal cohort consisted of 81 children with urine samples available in both periods. The reduction from the initial sample to the longitudinal cohort was primarily due to temporary absence, seasonal migration, or non-participation during one of the sampling rounds, which are common features of rural agricultural communities. Although detailed comparative information between included and excluded participants was not systematically available, no evidence suggested systematic differences in key exposure-related characteristics, such as residential proximity to treated agricultural fields, given the relatively homogeneous spatial configuration of the study setting.

The longitudinal within-subject design reduces the influence of between-subject variability, thereby strengthening the internal validity of temporal comparisons. Nonetheless, potential selection bias associated with loss to follow-up is acknowledged as a limitation.

A total of 168 urine samples were collected in March and 93 in December, reflecting partial participation across sampling rounds. The main longitudinal analyses were restricted to paired observations from the 81 children who participated in both sampling periods.

In the EM community (total population: 1399), the analytical cohort of 81 children aged 0–14 years was drawn from a finite population of 437 children (2020 Census). Under standard assumptions (95% confidence level, *p* = 0.5), this corresponds to an estimated margin of error of approximately 9.8%, accounting for finite population correction.

Although this level of precision may be considered modest, it is consistent with methodological standards for studies conducted in small rural populations, where population size constrains achievable samples [37].

The socio-economic structure of the community is characterised by a high proportion of economically active individuals engaged in waged agricultural labour. This reflects the local agrarian context and constitutes a defining characteristic of the population rather than a source of sampling bias.

### 2.2. Urinary Biomonitoring and Chemical Analysis

Urinary pesticide concentrations were determined using liquid chromatography coupled with tandem mass spectrometry (LC–MS/MS) using an Agilent 6460 Triple Quadrupole LC/MS system (Agilent Technologies, Santa Clara, CA, USA). Chromatographic separation was performed using a reverse-phase Zorbax Eclipse XDB-C18 column (Agilent Technologies, Santa Clara, CA, USA) under gradient elution conditions with water (0.1% formic acid) and acetonitrile (Sigma-Aldrich, St. Louis, MO, USA) as mobile phases. The injection volume was 5 µL, and the flow rate was maintained at 0.5 mL/min.

Quantification was performed using external calibration curves ranging from 0.01 to 1000 µg/L for each pesticide, with coefficients of determination (R^2^) greater than 0.99 for all analytes. The use of LC–MS/MS allowed for the detection of compounds with low UV absorbance, including macrocyclic lactones such as emamectin.

The analytical panel included pesticides commonly used in the study region, encompassing herbicides, insecticides, fungicides, and selected compounds. The selection of analytes was guided by a context-specific and transdisciplinary approach integrating socio-agronomic survey data, ethnographic fieldwork, and previous biomonitoring studies.

Because the analytical panel focused primarily on parent compounds, measured concentrations reflect recent or ongoing exposure rather than cumulative internal dose. Accordingly, detection patterns were interpreted as indicative of repeated exposure events in a context characterised by recurrent pesticide application.

Urinary concentrations were expressed in µg/L. Values below 0.0001 µg/L were considered below the limit of detection (LOD) and treated as left-censored data. When required, values below the LOD were imputed as LOD/√2, following standard practice [15,38].

A uniform detection threshold was applied across the analytical panel to ensure consistency in the treatment of low-level measurements. However, this approach may affect comparability between compounds, and detection frequencies should therefore be interpreted with caution.

### 2.3. Sample Handling and Analytical Quality Control

Urine samples were collected under standardised conditions in sterile polypropylene containers (Thermo Fisher Scientific, Waltham, MA, USA). Following collection, samples were kept refrigerated during transport and subsequently stored at −20 °C under controlled conditions until analysis.

Prior to analysis, urine samples underwent centrifugation to remove particulate matter, and when required, protein precipitation using an organic solvent. The resulting supernatant was transferred to vials for LC-MS/MS analysis.

All analyses were conducted in an accredited laboratory at the University of Guadalajara using validated procedures based on LC-MS/MS.

Quality assurance and quality control (QA/QC) procedures included the use of calibration curves, procedural blanks, duplicate samples, and verification standards to ensure analytical accuracy and precision. Instrument performance was regularly monitored throughout the analytical process, and internal standards were applied where appropriate.

### 2.4. Socio-Agronomic Questionnaire

The second methodological strategy involved the administration of a structured questionnaire to the heads of households of children participating in the biomonitoring component who consented to home visits. Between July and December 2018, a total of 72 questionnaires were completed, representing the majority of participating households. As the questionnaire was administered at the household level, a single questionnaire could correspond to more than one child included in the biomonitoring component. This accounts for the difference between the number of questionnaires completed and the total number of children included in the study.

The instrument collected information on household origin, principal economic activities of household heads and other family members, parental contact with pesticides, use of protective equipment during pesticide application, and family history of pesticide intoxication. For agricultural producers, additional information was obtained on cultivated land area, crop types, and pesticides applied during the previous agricultural cycle.

The questionnaire captured key socio-demographic, occupational, and agro-environmental characteristics relevant to pesticide exposure, including household composition, involvement in agricultural labour, proximity to cultivated fields, pesticide use practices, and water sources.

For the subset of children included in the analysis of agricultural versus non-agricultural households, questionnaire data were available for all cases and were used to classify household occupational status. The questionnaire was administered at the household level during the December field campaign, following urine sample collection, to ensure complete coverage of children participating in both sampling periods. Information obtained from each household was applied to all participating children within that household.

Questionnaire data were used to contextualise biomonitoring findings and to support the interpretation of exposure patterns. Rather than being treated as independent explanatory variables in statistical models, these data contributed to a transdisciplinary analytical framework integrating quantitative and qualitative evidence.

### 2.5. Ethnographic Component

The third strategy consisted of an ethnographic study conducted between 2018 and 2024 within the framework of an intervention-oriented project aimed at reducing children’s exposure to pesticides in El Mentidero. Fieldwork involved fortnightly visits of two days’ duration, during which systematic participant observation and detailed field notes were undertaken [39,40]. A total of 60 open-ended interviews were conducted with heads of household and local and municipal authorities, together with 28 focus group sessions involving farmers, mothers, primary and secondary school students, and teachers. These qualitative methods enabled the documentation of agricultural practices, household labour organisation, exposure conditions, use or absence of protective equipment, access to health services, and other contextual factors relevant to understanding children’s exposure.

Field observations also documented the spatial proximity between households and cultivated fields, with residential structures frequently located within a few metres of areas where pesticides are regularly applied.

### 2.6. Statistical Analysis

Comparisons between the two sampling rounds were conducted using intra-individual longitudinal analyses. Changes in pesticide detection frequency were assessed using McNemar’s test for paired binary data. Differences in urinary concentrations between sampling periods were evaluated using the Wilcoxon signed-rank test for paired urine samples. Longitudinal changes in detection probability were further analysed using generalised estimating equation (GEE) logistic models to account for intra-individual correlation across repeated measurements.

To evaluate the potential influence of urinary dilution, a sensitivity analysis was conducted using creatinine-adjusted concentrations (µg/g creatinine) in the subset of participants with available measurements at both sampling periods (*n* = 23). Because creatinine data were not available for the full cohort, these analyses were treated as complementary to the primary longitudinal analysis based on unadjusted concentrations. To control for the error associated with multiple comparisons across pesticides, *p*-values were adjusted using the False Discovery Rate (FDR) procedure proposed by Benjamini and Hochberg [41].

A detailed description of the statistical procedures, including paired longitudinal analyses, handling of censored data, and GEE models, as well as full quantitative results for all analyses (test statistics, *p*-values, effect sizes, and multiple comparison adjustments), is provided in the Statistical Supplementary Materials (Tables S1–S3).

Statistical analyses were performed using IBM SPSS Statistics version 26.0 (IBM Corp., Armonk, NY, USA). Figures were prepared using Microsoft Excel 365 (Microsoft Corp., Redmond, WA, USA).

### 2.7. Analytical Considerations and Limitations

Several analytical considerations should be acknowledged. First, although a consistent detection threshold (<0.0001 µg/L) was applied, compound-specific limits of detection (LOD) and quantification (LOQ) were not available for all analytes, which may limit comparability across compounds. Second, the analytical panel represents a targeted but non-exhaustive selection of pesticides relevant to the study context, reflecting a partial but contextually grounded exposure profile. Third, as the analysis focused on parent compounds, the results primarily reflect short-term exposure patterns rather than cumulative internal dose.

Despite these limitations, the integration of longitudinal biomonitoring with socio-agronomic and ethnographic data provides a robust framework for interpreting pesticide exposure patterns and their socio-environmental determinants. Although potential selection bias associated with loss to follow-up cannot be entirely excluded, the relatively homogeneous exposure context and the longitudinal within-subject design are expected to mitigate its impact. Additionally, the sensitivity analysis based on creatinine-adjusted concentrations was conducted on a relatively small subset of participants (*n* = 23) due to the limited availability of creatinine measurements across both sampling periods. This reduced sample size limits the statistical strength of the adjusted analysis, and the results should therefore be interpreted with caution. However, the consistency between adjusted and unadjusted findings provides additional support for the robustness of the observed exposure patterns.

## 3. Results

### 3.1. Characteristics of the Longitudinal Cohort

A total of 168 urine samples were collected in March and 93 in December 2018, reflecting partial participation across sampling rounds. The analytical longitudinal cohort consisted of 81 children with urine samples available in both periods. The sex distribution was balanced (51.9% boys and 48.1% girls).

Age distribution is presented in Table 1. The largest proportion corresponded to children aged 9–11 years (33.3%), followed by those aged 6–8 years (29.6%), 12–14 years (22.2%), and 3–5 years (14.8%).

### 3.2. Detection Frequency of Pesticides

Detection frequencies for all analysed pesticides are presented in Table 2. Three compounds—emamectin, methomyl, and parathion—were detected in all samples across both sampling periods (100% detection). While this pattern is consistent with widespread exposure, it should be interpreted in light of the uniform detection threshold applied across the analytical panel, which may influence comparability with other compounds.

**Figure 2:**
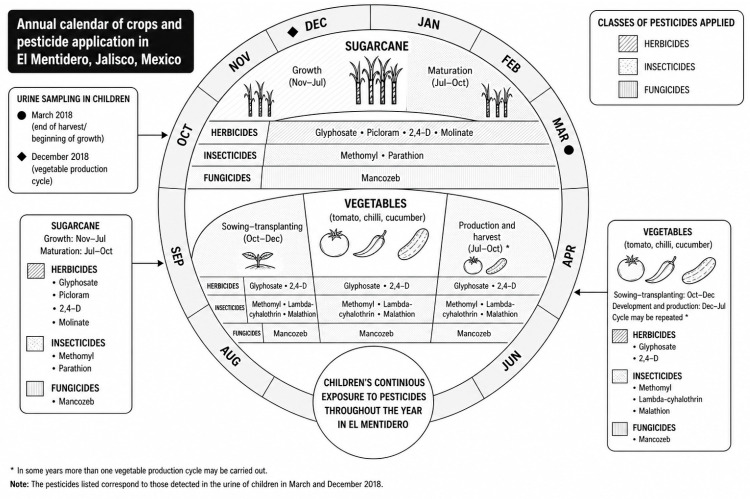
Annual crop cycle and pesticide application calendar in El Mentidero, Jalisco, Mexico, including the timing of urine sampling in March and December 2018. Note. The diagram illustrates the temporal distribution of pesticide use across dominant crops and its alignment with the timing of biomonitoring campaigns. Source: Author’s own elaboration based on fieldwork, biomonitoring data, and socio-agronomic information.

Other compounds showed lower detection frequencies, generally below 10%. Some pesticides were detected in only one sampling period. For example, lambda-cyhalothrin and malathion were detected in March but not in December. The temporal distribution of agricultural activities and pesticide application periods in the study area, together with the timing of the biomonitoring campaigns, is summarised in Figure 2.

### 3.3. Longitudinal Changes in Urinary Concentrations

**Figure 3:**
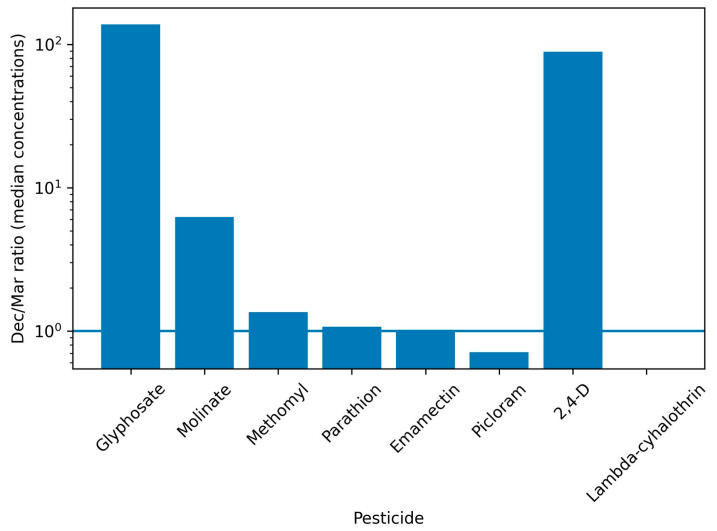
Relative change in median urinary pesticide concentrations between March and December 2018 (December/March ratio) among children with paired observations in El Mentidero, Jalisco, Mexico. Note. Values greater than 1 indicate higher median concentrations in December, whereas values close to 1 indicate stability across sampling periods. Ratios are calculated from median concentrations reported in Supplementary Table S2.

Figure 3 presents the ratio of median urinary pesticide concentrations measured in December relative to March (Dec/Mar) among children with paired observations. This summary measure complements the paired statistical analyses by providing a comparative visual representation of the direction and magnitude of change across compounds. A logarithmic scale was used to improve visual comparability across compounds with different magnitudes of change.

Paired concentration analyses revealed compound-specific longitudinal changes. Glyphosate showed a marked increase between March and December among children with paired non-missing values (*n* = 76), with median concentrations rising from 0.028 µg/L (IQR: 0.007–1.914) to 2.801 µg/L (IQR: 2.189–3.630). Overall, 65 out of 76 children (85.5%) showed increased concentrations, while 11 (14.5%) showed decreases. The Wilcoxon signed-rank test indicated a statistically significant increase (W = 272, *p* < 0.001).

Similar increases were observed for molinate and methomyl. Molinate increased from a median of 0.009 µg/L (IQR: 0.004–0.019) in March to 0.055 µg/L (IQR: 0.031–0.086) in December (*n* = 78), with increases observed in 72 children (92.3%; W = 122, *p* < 0.001). Methomyl increased from 0.031 µg/L (IQR: 0.030–0.031) to 0.041 µg/L (IQR: 0.038–0.048), with all 81 children showing higher concentrations in December (W = 0, *p* < 0.001). Parathion also showed a statistically significant increase, whereas emamectin remained stable and picloram decreased significantly. Full quantitative results, including sample sizes, medians, interquartile ranges, proportions of increases and decreases, Wilcoxon statistics, *p*-values, and effect sizes, are reported in the Statistical Supplementary Materials. Results from the creatinine-adjusted sensitivity analysis are also presented therein.

### 3.4. Comparison by Household Occupational Activity

Detection frequencies and mean concentrations were compared between children from agricultural and non-agricultural households (Table 3). Analyses were restricted to children with paired samples and valid household classification.

For most pesticides, no consistent differences in detection frequencies were observed between groups. For glyphosate and 2,4-D, differences between groups were observed in some sampling periods; however, these patterns were not consistent across both rounds. For emamectin, methomyl, and parathion, detection was 100% in both groups and across both sampling periods.

Overall, detection patterns were similar between children from agricultural and non-agricultural households for most compounds.

### 3.5. Sensitivity Analysis: Creatinine Adjustment

A sensitivity analysis using creatinine-adjusted concentrations (*n* = 23) was conducted to assess the potential influence of urinary dilution. Results were consistent with those obtained using unadjusted concentrations, indicating that urinary dilution did not substantially affect the observed exposure patterns.

## 4. Discussion

### 4.1. Pesticide Exposure Among Children Living in Agricultural Landscapes

The results of this study demonstrate widespread exposure to multiple pesticides among children living in an agricultural community characterised by intensive crop production and close spatial proximity between residential areas and cultivated fields. In this context, children’s everyday environments are shaped by the continuous presence of pesticides across domestic, school, and community spaces, reflecting a form of exposure that is embedded and normalised within daily life rather than limited to discrete events. Biomonitoring results revealed both persistent detection of several compounds and temporal variation in urinary concentrations, indicating that children are exposed to pesticide mixtures through ongoing and overlapping processes rather than isolated exposure events.

These findings are consistent with previous biomonitoring studies conducted in agricultural settings, which have reported similar patterns of pesticide detection and seasonal variation. In the present study, this pattern is reflected in the observed longitudinal increases in specific compounds, supporting the interpretation of exposure as temporally linked to agricultural cycles. Studies conducted in Europe and Latin America have reported similar patterns of detection and exposure intensity, with concentrations varying according to seasonal agricultural activity and proximity to treated fields [7,42,43,44]. In addition, recent cohort studies suggest that even low-level, chronic exposure to non-persistent pesticides may be associated with developmental effects [3]. Although direct comparisons across studies are methodologically complex, the patterns observed here are broadly consistent with this international evidence base and support calls to re-examine current assumptions regarding “acceptable” exposure levels in children.

It is important to note that health-based reference values, such as reference doses (RfD), are typically derived from external exposure estimates and are not directly applicable to urinary concentrations of parent compounds. Accordingly, the values reported in this study should be interpreted as indicators of exposure rather than direct measures of internal dose or health risk. Nonetheless, the widespread and repeated detection of multiple pesticides, particularly those with known toxicological profiles, underscores the public health relevance of these findings.

Recent studies conducted in Latin America and other regions of the Global South have increasingly documented patterns of pesticide exposure among children that cannot be fully explained by occupational or take-home pathways alone. Biomonitoring research in agricultural communities has shown that children residing in close proximity to treated fields may exhibit detectable levels of pesticide residues irrespective of direct household involvement in agricultural labour [5,45]. These findings suggest that environmental dispersion processes, spatial proximity, and shared community environments play a central role in shaping exposure patterns. However, despite this growing body of evidence, relatively few studies have explicitly conceptualised these dynamics as community-level exposure processes, particularly in rural settings of the Global South. In this context, the present study contributes to bridging this gap by integrating biomonitoring, socio-agronomic, and ethnographic approaches to examine pesticide exposure as a territorially embedded and structurally mediated phenomenon.

From a toxicological perspective, these findings should be interpreted in light of emerging evidence on the mechanisms underlying pesticide toxicity. Recent studies indicate that health effects may be mediated not only by active ingredients but also by complex mixtures, including co-formulants that can enhance toxicity and bioavailability [9]. In addition, increasing concern has been raised regarding the neurodevelopmental effects of newer pesticide classes, such as neonicotinoids, which may interfere with critical stages of brain development [10]. While the present study does not estimate internal dose or risk, this evolving toxicological evidence supports the interpretation of the findings as indicative of potentially harmful exposure contexts. Accordingly, a precautionary perspective is warranted, particularly for vulnerable populations such as children.

These results also support the relevance of a community-level exposure framework. Rather than being limited to occupational or individual behaviours, pesticide exposure in this context appears to be shaped by broader territorial dynamics, including proximity to agricultural fields, patterns of pesticide application, and environmental dispersion processes. This interpretation is consistent with recent conceptual and empirical work emphasising that exposure in rural settings is socially and spatially structured [12,13]. From this perspective, children’s exposure reflects not only environmental contamination but also underlying conditions of environmental inequality, in which certain populations bear a disproportionate burden of risk [14]. These findings highlight the need to move beyond individual-level explanations and toward integrative approaches that address the structural and territorial determinants of exposure.

The universal detection of emamectin, methomyl, and parathion across both sampling periods indicates sustained environmental presence of these compounds. This pattern is consistent with continuous environmental contamination associated with recurrent pesticide application and supports the interpretation of persistent exposure at the community level. However, this finding should be interpreted with caution given the use of a uniform detection threshold across analytes, which may affect comparability between compounds.

Importantly, the findings reported here are consistent with previous biomonitoring studies conducted in agricultural communities in Mexico and the region of study, which have documented detectable levels of multiple pesticide residues in children and adolescents [5]. This continuity suggests that the patterns observed are not isolated, but rather indicative of persistent exposure conditions in rural agricultural regions of the country. By situating the present results within this national context, the study contributes to a growing body of evidence highlighting the need for strengthened regulatory frameworks, improved monitoring systems, and targeted public health interventions aimed at reducing pesticide exposure in vulnerable populations.

Longitudinal variation in urinary concentrations further supports this interpretation. The observed increases in certain compounds between sampling periods are consistent with seasonal agricultural dynamics, including crop maintenance, pest control, and harvest cycles. Similar patterns have been documented in biomonitoring studies where pesticide exposure is closely linked to the timing of agricultural activities [46,47,48].

Taken together, these findings indicate that children in this locality are subject to continuous environmental exposure shaped by agricultural production cycles, rather than discrete exposure events.

### 4.2. Beyond the Take-Home Pathway: Multiple Exposure Routes

Previous research has frequently emphasised occupational pathways, particularly the take-home mechanism, as a primary route of pesticide exposure among children. While this pathway is well-documented [21,22], the findings of this study suggest that it does not fully explain the observed exposure patterns.

The comparison between children from agricultural and non-agricultural households showed no consistent differences in detection frequencies for most pesticides. These results suggest that exposure is not restricted to households directly engaged in agricultural labour.

Instead, the results support the existence of multiple, simultaneous exposure processes, including environmental dispersion, household contamination, and potential dietary intake. This aligns with previous studies showing that children living in agricultural regions may experience significant pesticide exposure regardless of parental occupation [19,20,49].

In this context, pesticide exposure should be understood as a distributed and community-wide phenomenon, rather than one confined to specific occupational groups.

### 4.3. Environmental Dispersion and Spatial Configuration

The spatial configuration of the study area plays a central role in shaping exposure patterns. Residential areas, schools, and public spaces are located in very close proximity to agricultural fields, in some cases within only a few metres and rarely exceeding distances of a few tens of metres. This spatial arrangement effectively embeds everyday life within zones of pesticide application, creating conditions in which environmental dispersion processes directly affect inhabited spaces.

Under these conditions, pesticides may disperse beyond the site of application through spray drift, volatilisation, runoff, and the resuspension of contaminated soil and dust [50]. These processes contribute to the contamination of shared environments and increase the likelihood of indirect exposure.

Environmental monitoring studies have shown that pesticide residues can accumulate in soil, house dust, and indoor surfaces in homes located near treated fields [51,52], reinforcing the role of the domestic environment as an interface of exposure.

Children are particularly vulnerable to these pathways due to behavioural factors such as frequent hand-to-mouth activity and close contact with soil and dust [16,17].

### 4.4. Agricultural Production Systems and Temporal Dynamics

The exposure patterns observed must also be understood in relation to the structure of local agricultural production systems. The coexistence of sugarcane cultivation and intensive horticultural production generates overlapping cycles of pesticide application across the territory.

Because these crops require agrochemical inputs at different stages of the year, pesticide use is temporally distributed rather than concentrated in a single season, resulting in a continuous environmental presence reflected in both persistent detection and temporal variation in urinary concentrations.

This interpretation is consistent with findings from other agricultural regions, where seasonal exposure patterns are closely linked to crop cycles and pesticide application schedules [20,48], reinforcing the structural nature of exposure dynamics.

### 4.5. Implications for Environmental Health Research

These findings indicate that pesticide exposure cannot be adequately understood through single-pathway models focused exclusively on occupational or take-home mechanisms.

Instead, exposure should be conceptualised as a multi-scalar and territorially embedded process, shaped by the interaction between agricultural production systems, environmental dispersion, and settlement patterns. This perspective calls for integrated approaches that combine biomonitoring, environmental assessment, and spatial analysis in order to better capture the complexity of exposure pathways.

From a public health standpoint, interventions limited to workplace safety are unlikely to be sufficient. Reducing children’s exposure in such contexts requires addressing environmental sources of contamination at the community level, including land-use regulation, the establishment of buffer zones, and stricter control of pesticide application in proximity to residential areas.

These limitations reflect a broader structural condition: in settings where residential spaces are located in close proximity to intensively treated agricultural fields, exposure is not confined to specific activities but is embedded within the spatial organisation of everyday life. Under these conditions, proximity operates as a defining feature of a community-level exposure regime, in which environmental contamination circulates across shared spaces and affects the entire population irrespective of occupational status.

Addressing this challenge requires structural responses that reconnect health, territory, and food systems. The findings support the need for transitions toward agroecological production models that reduce reliance on hazardous chemical inputs and address the underlying drivers of exposure [53,54]. Such transitions are also closely linked to broader processes of environmental justice in rural communities where exposure is unevenly distributed and structurally produced [55].

## 5. Conclusions

This study provides evidence that pesticide exposure among children in intensively cultivated rural settings cannot be adequately understood through individual or occupational sources alone. The longitudinal biomonitoring results, combined with socio-agronomic and ethnographic evidence, indicate that exposure operates as a community-level exposure regime, in which multiple exposure processes converge and are continuously reproduced within the same territorial space.

Rather than reflecting isolated or episodic events, the observed exposure patterns point to processes that are structurally produced through agricultural systems characterised by intensive chemical inputs, labour dynamics, and regulatory limitations. These processes are territorially embedded in landscapes where close spatial proximity between residential areas and cultivated fields facilitates the circulation and persistence of pesticide residues across domestic, school, and community environments.

Within this context, the absence of statistically significant differences between children from agricultural and non-agricultural households should be interpreted cautiously. While consistent with a shared exposure environment, this finding also reflects the sociohistorical configuration of the locality, where migrant agricultural labour has shaped settlement patterns and blurred conventional occupational distinctions. In such settings, exposure is not confined to specific activities but is distributed across the community as a whole.

The transdisciplinary approach adopted in this study allows for a more comprehensive interpretation of these dynamics. By integrating biomonitoring data with ethnographic and socio-agronomic insights, it becomes possible to move beyond reductionist frameworks and to understand exposure as a socially and environmentally mediated process that unfolds across multiple scales.

From a public health perspective, these findings underscore the limitations of risk models that focus exclusively on individual behaviour or occupational exposure. Addressing pesticide exposure in rural communities requires interventions that engage with the structural and territorial conditions that sustain it, including land-use planning, regulation of pesticide application near residential areas, and the promotion of alternative agricultural practices.

In this sense, pesticide exposure should be understood not only as an environmental health issue but as a structurally produced and territorially embedded condition shaped by broader socio-environmental arrangements. Addressing this challenge requires interventions that go beyond individual risk mitigation and instead engage with the underlying configurations of agricultural production, land use, and environmental regulation. Such an approach is essential for effectively protecting children living in agricultural landscapes.

## Figures and Tables

**Table 1:** Distribution of children with samples in both screening rounds by age group.

Age Group (years)	Frequency	Percentage (%)
3–5	12	14.8
6–8	24	29.6
9–11	27	33.3
12–14	18	22.2
**Total**	**81**	**100**

**Table 2:** Frequency of pesticide detection among children with paired urine samples across two sampling rounds (March and December 2018).

Pesticide	Detected March *n* (%)	Detected December *n* (%)	Absolute Change (Percentage Points)
Acetochlor	6 (7.4)	5 (6.2)	−1.2
Ametryn	4 (4.9)	3 (3.7)	−1.2
Atrazine	8 (9.9)	6 (7.4)	−2.5
Carbendazim	7 (8.6)	6 (7.4)	−1.2
Carbofuran	3 (3.7)	2 (2.5)	−1.2
Diazinon	5 (6.2)	4 (4.9)	−1.3
Dimethoate	2 (2.5)	1 (1.2)	−1.3
Emamectin	81 (100.0)	81 (100.0)	0
Glyphosate	41 (50.6)	38 (46.9)	−3.7
Imazalil	4 (4.9)	0 (0.0)	−4.9
Lambda-cyhalothrin	9 (11.1)	0 (0.0)	−11.1
Malathion	7 (8.6)	0 (0.0)	−8.6
Methomyl	81 (100.0)	81 (100.0)	0
Molinate	2 (2.5)	1 (1.2)	−1.3
Parathion	81 (100.0)	81 (100.0)	0
Picloram	3 (3.7)	2 (2.5)	−1.2
Pyraclostrobin	3 (3.7)	2 (2.5)	−1.2
Thiabendazole	6 (7.4)	4 (4.9)	−2.5
2,4-D	5 (6.2)	4 (4.9)	−1.3

Note: Percentages were calculated based on the total number of children included in the longitudinal analysis (*n* = 81).

**Table 3:** Detection frequencies and mean concentrations of selected pesticides among children from agricultural and non-agricultural households (March and December 2018).

Pesticide	Month	Agricultural Households (*n* = 52) *n* (%)	Non-Agricultural Households (*n* = 28) *n* (%)	Mean (µg/L) Agricultural	Mean (µg/L) Non-Agricultural
Glyphosate	March	28 (53.8)	13 (46.4)	1.119	0.905
Glyphosate	December	25 (48.1)	13 (46.4)	1.37	1.402
2,4-D	March	3 (5.8)	2 (7.1)	0.012	0.008
2,4-D	December	3 (5.8)	1 (3.6)	0.012	0.014
Emamectin	March	52 (100) **	28 (100) **	0.065	0.056
Emamectin	December	52 (100) **	28 (100) **	0.056	0.056
Methomyl	March	52 (100) **	28 (100) **	0.031	0.031
Methomyl	December	52 (100) **	28 (100) **	0.043	0.044
Parathion	March	52 (100) **	28 (100) **	0.062	0.062
Parathion	December	52 (100) **	28 (100) **	0.068	0.067
Molinate	March	1 (1.9)	1 (3.6)	0.011	0.035
Molinate	December	1 (1.9)	0 (0.0)	0.069	0.053
Picloram	March	2 (3.8)	1 (3.6)	0.122	0.13
Picloram	December	1 (1.9)	1 (3.6)	0.104	0.134

Note: Values represent the number and percentage of children with detectable concentrations of each pesticide within each subgroup. Percentages are calculated based on the total number of children in each household category. The analysis only included children with paired urine samples in both sampling rounds. One child from the longitudinal cohort was excluded from the household-stratified analysis because inconsistent information on household occupational status precluded reliable classification; therefore, this analysis was based on 80 children. ** Detection frequencies of 100% indicate that the pesticide was detected in all analysed samples within the subgroup.

## Data Availability

The data presented in this study are not publicly available due to ethical and privacy restrictions related to research involving minors and sensitive health information. Data may be made available from the corresponding author upon reasonable request and subject to approval by the relevant ethics committee.

## Supplementary Materials

The following supporting information can be downloaded at: https://www.mdpi.com/article/10.3390/ijerph23050664/s1, Table S1. Detection frequencies in paired urine samples; Table S2. Changes in urinary pesticide concentrations between March and December 20 Benjamini–Hochberg procedure 18 (paired Wilcoxon signed-rank analysis); Table S3. Longitudinal patterns in pesticide detection (GEE models).

## Abbreviation

The following abbreviation is used in this manuscript:

EM	El Mentidero
