# Supplementary material for: Beyond the Take-Home Pathway: Community-Level Pesticide Exposure Among Children Living in an Intensively Cultivated Agricultural Landscape
Source: Int J Environ Res Public Health. 2026 May 18;23(5):664. doi: 10.3390/ijerph23050664 (PMC13206163; doi:10.3390/ijerph23050664)
Supplement: Supplementary file 1 [file ijerph-23-00664-s001.zip › ijerph-4260185-supplementary.pdf]

Longitudinal statistical analysis of urinary pesticide exposure Appendix

Study design and analytical framework

This analysis was conducted using paired observations, with values below the analytical detection threshold imputed as LOD/√2 to allow inclusion of low-level measurements while preserving comparability across sampling periods. Longitudinal designs with repeated biological measurements are widely used in environmental biomonitoring because they enable the assessment of temporal changes in exposure while reducing inter-individual variability related to physiological and behavioural differences between participants (Barr et al., 2005).

The analytical strategy followed two complementary components: (i) analysis of detection frequencies (detected vs. non-detected); and (ii) analysis of concentration changes among paired urine samples. This dual approach is commonly used in biomonitoring studies where a proportion of measurements may fall near or below analytical detection limits.

The supplementary tables provide full quantitative statistical results for all analyses conducted in this study, including test statistics, p-values, effect sizes, and multiple comparison corrections (false discovery rate, FDR). These results are presented to ensure full transparency and reproducibility of the analytical approach.

Analysis of changes in detection frequency

Changes in detection frequency between the two sampling rounds were evaluated using McNemar's test, which is appropriate for paired binary data.

McNemar's test evaluates whether the probability of detection differs between two repeated measurements within the same individuals by comparing discordant pairs (i.e., cases where detection status differs between sampling rounds). This approach allows the assessment of directional changes in pesticide detection probability over time.

Supplementary Table S1. Detection frequencies in paired urine samples

Pesticide	Detected March n (%)	Detected December n (%)	Absolute change (%)
Acetochlor	6 (7.4)	5 (6.2)	−1.2
Ametryn	4 (4.9)	3 (3.7)	−1.2
Atrazine	8 (9.9)	6 (7.4)	−2.5
Carbendazim	7 (8.6)	6 (7.4)	−1.2
Carbofuran	3 (3.7)	2 (2.5)	−1.2
Diazinon	5 (6.2)	4 (4.9)	−1.3
Dimethoate	2 (2.5)	1 (1.2)	−1.3
Emamectin	81 (100)	81 (100)	0
Glyphosate	41 (50.6)	38 (46.9)	−3.7
Imazalil	4 (4.9)	0 (0)	−4.9
Lambda-cyhalothrin	9 (11.1)	0 (0)	−11.1
Malathion	7 (8.6)	0 (0)	−8.6
Methomyl	81 (100)	81 (100)	0
Molinate	2 (2.5)	1 (1.2)	−1.3
Parathion	81 (100)	81 (100)	0
Picloram	3 (3.7)	2 (2.5)	−1.2
Pyraclostrobin	3 (3.7)	2 (2.5)	−1.2
Thiabendazole	6 (7.4)	4 (4.9)	−2.5
2,4-D	5 (6.2)	4 (4.9)	−1.3

Note. Detection frequencies were calculated for paired urine samples from 81 children participating in both sampling rounds.

Analysis of changes in urinary concentrations

Differences in urinary pesticide concentrations between the two sampling rounds were evaluated using the paired Wilcoxon signed-rank test.

Urinary pesticide concentrations typically show right-skewed distributions and may include extreme values. For this reason, analyses were performed on log-transformed concentrations. The Wilcoxon signed-rank test is a non-parametric method appropriate for paired urine samples when normality cannot be assumed.

This analysis was restricted to observations with detectable concentrations in both sampling rounds.

Supplementary Table S2. Changes in urinary pesticide concentrations between March and December 2018 (paired Wilcoxon signed-rank analysis)

Pesticide	n (paired)	Median March (µg/L)	Median December (µg/L)	IQR March (µg/L)	IQR December (µg/L)	Dec/Mar ratio	Increase n (%)	Decrease n (%)	Stable n (%)	Wilcoxon W	p-value	FDR-adjusted p-value	Wilcoxon r
Acetochlor	81	0.00007	0.00007	0.00007–0.00007	0.00007–0.00007	1.00	0 (0.0)	0 (0.0)	81 (100.0)	NA	NA	NA	NA
Ametryn	81	0.00007	0.00007	0.00007–0.00007	0.00007–0.00007	1.00	0 (0.0)	0 (0.0)	81 (100.0)	NA	NA	NA	NA
Atrazine	81	0.00007	0.00007	0.00007–0.00007	0.00007–0.00007	1.00	0 (0.0)	0 (0.0)	81 (100.0)	NA	NA	NA	NA
Carbendazim	81	0.00007	0.00007	0.00007–0.00007	0.00007–0.00007	1.00	0 (0.0)	0 (0.0)	81 (100.0)	NA	NA	NA	NA
Carbofuran	81	0.00007	0.00007	0.00007–0.00007	0.00007–0.00007	1.00	0 (0.0)	0 (0.0)	81 (100.0)	NA	NA	NA	NA
Diazinon	81	0.00007	0.00007	0.00007–0.00007	0.00007–0.00007	1.00	0 (0.0)	0 (0.0)	81 (100.0)	NA	NA	NA	NA
Dimethoate	81	0.00007	0.00007	0.00007–0.00007	0.00007–0.00007	1.00	0 (0.0)	0 (0.0)	81 (100.0)	NA	NA	NA	NA
Emamectin	81	0.056	0.056	0.056–0.056	0.056–0.056	1.00	40 (49.4)	41 (50.6)	0 (0.0)	1557	0.626	0.626	-0.054
Glyphosate	80	0.021	2.866	0.006–1.848	2.189–3.729	138.10	69 (86.2)	11 (13.8)	0 (0.0)	190	<0.001	<0.001	0.767
Imazalil	81	0.00007	0.00007	0.00007–0.00007	0.00007–0.00007	1.00	0 (0.0)	1 (1.2)	80 (98.8)	0	0.317	0.353	0.000
Lambda-cyhalothrin	81	0.029	0.00007	0.019–0.048	0.00007–0.00007	0.00	0 (0.0)	81 (100.0)	0 (0.0)	0	<0.001	<0.001	-0.869
Malathion	81	0.00007	0.00007	0.00007–0.00007	0.00007–0.00007	1.00	0 (0.0)	5 (6.2)	76 (93.8)	0	0.043	0.054	0.000
Methomyl	81	0.030	0.041	0.030–0.031	0.038–0.048	1.35	81 (100.0)	0 (0.0)	0 (0.0)	0	<0.001	<0.001	0.869
Molinate	81	0.009	0.053	0.003–0.019	0.031–0.083	6.25	75 (92.6)	6 (7.4)	0 (0.0)	140	<0.001	<0.001	0.795
Parathion	81	0.062	0.066	0.062–0.062	0.064–0.069	1.07	81 (100.0)	0 (0.0)	0 (0.0)	0	<0.001	<0.001	0.869
Picloram	81	0.061	0.044	0.039–0.119	0.032–0.064	0.71	27 (33.3)	54 (66.7)	0 (0.0)	1064	0.005	0.007	-0.312
Pyraclostrobin	81	0.00007	0.00007	0.00007–0.00007	0.00007–0.00007	1.00	0 (0.0)	0 (0.0)	81 (100.0)	NA	NA	NA	NA
Thiabendazole	81	0.00007	0.00007	0.00007–0.00007	0.00007–0.00007	1.00	0 (0.0)	0 (0.0)	81 (100.0)	NA	NA	NA	NA
2,4-D	81	0.00007	0.006	0.00007–0.003	0.00007–0.014	88.55	49 (60.5)	14 (17.3)	18 (22.2)	263	<0.001	<0.001	0.643

Note . Values below the analytical detection threshold (<0.0001 µg/L) were imputed as LOD/√2 for concentration analyses. Wilcoxon signed-rank tests were applied to log-transformed paired concentrations. W, p-values and Wilcoxon r are reported only when within-pair variability was present. NA indicates that the test was not applicable because all paired differences were zero after imputation. Positive r values indicate higher concentrations in December; negative r values indicate lower concentrations in December. Effect size interpretation: small ≈0.10, medium ≈0.30, large ≥0.50. FDR-adjusted p-values were calculated using the Benjamini–Hochberg procedure. For compounds with uniformly imputed values across both sampling periods, observed stability reflects the absence of detectable

concentrations rather than true biological invariance. For emamectin, small within-individual fluctuations around a stable central tendency resulted in both increases and decreases without statistically significant change.

Technical clarification. Parathion showed real within-child variation, with all paired observations increasing from March to December. Emamectin also showed small within-child variation, but the change was not statistically significant. For compounds with identical imputed values in both periods for all children, the Wilcoxon test and r effect size are reported as NA because there was no within-pair variability to test.

Longitudinal modelling using generalised estimating equations (GEE)

To complement the paired analyses, generalised estimating equation (GEE) logistic models were used to assess longitudinal changes in pesticide detection probability while accounting for within-subject correlation between repeated measurements.

GEE models provide population-averaged estimates and are widely used in epidemiological studies with repeated measures because they account for correlated observations within individuals over time.

Supplementary Table S3. Longitudinal patterns in pesticide detection (GEE models)

**Pesticide**	**Pattern of change**
Glyphosate	Slight increase in detection probability
Molinate	Increase
Methomyl	Stable
Parathion	Persistent detection
Emamectin	Persistent detection

Note. Analyses were restricted to paired observations with detectable concentrations in both sampling periods. Concentration differences were evaluated using the Wilcoxon signed-rank test applied to log-transformed values. The Dec/Mar ratio represents the relative change in median concentration between sampling periods. "Increase" and "Decrease" indicate the number and proportion of children showing higher or lower concentrations in December relative to March. "ns" indicates non-significant differences.

Adjustment for urinary dilution

To evaluate the potential influence of urinary dilution, a sensitivity analysis using creatinine-adjusted concentrations (µg/g creatinine) was performed in the subset of participants with creatinine measurements available in both sampling rounds.

Creatinine adjustment is commonly used in urinary biomonitoring studies to reduce variability related to urine dilution (Barr et al., 2005).

References

Benjamini, Y., & Hochberg, Y. (1995). Controlling the false discovery rate: A practical and powerful approach to multiple testing. *Journal of the Royal Statistical Society: Series B*, 57(1), 289–300.
